# Azanucleoside treatment leads to B-cell precursor acute lymphoblastic leukemia^∗^

**DOI:** 10.1016/j.bneo.2025.100161

**Published:** 2025-08-19

**Authors:** Vijay Negi, Ryan Bertoli, Olivia Tuckey, Yuelin Jack Zhu, Robert L. Walker, Michael J. Difilippantonio, James H. Doroshow, Paul S. Meltzer, Peter D. Aplan

**Affiliations:** 1 Genetics Branch, Center for Cancer Research, National Cancer Institute, National Institutes of Health, Bethesda, MD; 2 Division of Cancer Treatment and Diagnosis, National Cancer Institute, National Institutes of Health, Bethesda, MD; 3 Myeloid Malignancies Program, National Institutes of Health, Bethesda, MD

## Abstract

5-Aza-4'-thio-2'-deoxycytidine (ATC) is an azanucleoside cytidine analog under investigation in preclinical studies for solid tumors as a promising DNA methyltransferase 1 (DNMT1) inhibitor. Repeated treatment with ATC has previously been shown to induce acute lymphoblastic leukemia (ALL) of both B-cell and T-cell origin in mice. Herein, RAG-1 deficient or “knockout” (KO) mice (B6.129S7-RAG-1tm1Mom/J) were treated with ATC to determine if ATC could be oncogenic in nonlymphoid cells. However, ATC treatment targeted early B progenitors and invariably led to B-lineage ALL, with a gene expression signature similar to human B-cell precursor (BCP)-ALL. Whole-exome sequencing revealed numerous single base substitutions of cytosine, primarily C>G transversions at CpG dinucleotides, within genes important for BCP-ALL. Bisulfite sequencing and treatment with a noncovalent DNMT1 inhibitor indicated that methylated cytosines were preferred targets for mutagenesis. This study reveals that ATC exposure leads to both DNMT1-dependent and -independent mutagenesis and provides a direct link between ATC exposure, a complex mutational signature, and malignant transformation.

## Key Points

• Treatment with ATC, an investigational azanucleoside, invariably leads to BCP-ALL in immunodeficient mice.
• Two related mechanisms result in C>G mutation involving known cancer genes; the primary mechanism mutates 5-methyl-cytosine in a CpG context.

## Visual Abstract

**Figure:**
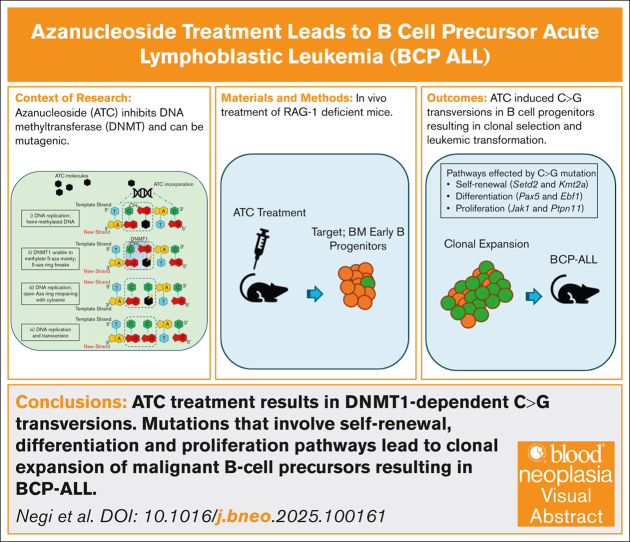

## Introduction

DNA methylation is an epigenetic mechanism in which a methyl group is transferred from S-adenyl methionine to the C5 position of cytosine to form 5-methylcytosine.^1^ This process is mediated by a family of enzymes known as DNA methyltransferases (DNMTs).^1^ In general, DNMT3A and DNMT3B function as de novo methylases that establish methylation patterns during embryogenesis, whereas DNMT1 recognizes hemi-methylated DNA and is considered the principal maintenance methyltransferase, responsible for preserving DNA methylation marks during cell division.^2^^,^^3^

Although DNA methylation is a normal biological process, many cancers, including hematologic malignancies such as myelodysplastic syndrome and acute myeloid leukemia (AML), have been linked to hypermethylation of cytosine residues.^4^, ^5^, ^6^ It has been proposed that this hypermethylation may lead to silencing and functional inactivation of tumor suppressor genes.^7^ This concept led to the development of DNMT1 inhibitors (DNMT1i), based on the premise that inhibition of DNMT1 mediated methylation may reactivate tumor suppressor genes that have been silenced by methylation.^8^, ^9^, ^10^, ^11^ The most common approach to inhibit DNMT1 is through the use of cytidine analogs that deplete DNMT1 and thereby inhibit DNA methylation.^12^ Two such cytidine analogs, 5-aza-2'-deoxycytidine (decitabine) and 5-azacytidine, have been approved by the U. S. Food and Drug Administration and European Medicines Agency for the treatment of myelodysplastic syndromes, chronic myelomonocytic leukemia, and AML.^13^^,^^14^ 5-aza-4'-thio-2'-deoxycytidine (aza-TdCyd or ATC) is a recently developed cytidine analog which has been used in preclinical studies against solid tumors as a promising DNMT1i.^15^^,^^16^

A recent report demonstrated that ATC has mutagenic and carcinogenic properties leading to lymphoid leukemia in mice.^17^ The leukemias that developed were primarily precursor T-cell leukemia/lymphoma (pre–T-LBL or T-ALL), and less commonly B-cell precursor acute lymphoblastic leukemia (BCP-ALL). Most (>80%) acquired mutations in the leukemic mice were C>G transversions, primarily in a specific 5'-NCG-3' context.^17^ The observation that the malignancies developing in ATC-treated mice were invariably of lymphoid origin and arose early in life (<7 months of age) led to the hypothesis that if ATC-treated mice lived longer, they might develop nonlymphoid malignancies with the same unique mutational signature. That is, if mice were “protected” from developing lymphoid malignancies, this might uncover the potential for solid tumors with characteristic C>G transversions later in life. To study this question, we treated RAG-1 deficient mice^18^ with ATC, reasoning that they would be protected from B and T-cell malignancies because they lack mature B or T cells.

## Materials and methods

Please see the online supplemental Methods for detailed description of experimental procedures.

### Mouse strain

RAG-1 deficient or “knockout” (KO) mice (B6.129S7-RAG-1tm1Mom/J) were purchased from The Jackson Laboratory. All animal experiments were approved by the National Cancer Institute Intramural Animal Care and Use Committee and maintained in a National Institutes of Health intramural animal facility.

### Drug treatment and schedule

5-Aza-4'-thio-2'-deoxycytidine (Aza-TdCyd, ATC, NSC777586, or CAS 169514-76-5) was obtained from the Drug Synthesis and Chemistry Branch, Developmental Therapeutics Program (RRID:SCR_003057), Division of Cancer Treatment and Diagnosis, National Cancer Institute.

### Bulk RNA sequencing and GSEA

Bone marrow (BM) and spleen samples from leukemic RAG-1 KO mice samples, as well as sorted RAG-1 KO BM samples, were used for total RNA isolation. Hierarchical clustering analysis was performed using R statistical software (v 4.4.1) and RStudio (v 2023.12.0.369) with base R functions. Gene set enrichment analysis (GSEA) analysis was performed using GSEA software (Broad Institute v 4.3.2) and compared to gene sets curated from publicly available murine B lineage ALL data (GSE221597) and human B lineage ALL data (GSE79533).

### WES

Whole-exome sequencing (WES) was performed on RAG-1 KO leukemia samples or BCP-ALL cell line (T259) samples. For mutational signature analysis, somatic variants were further filtered by the following criteria: baitRegion = “TRUE,” FILTER = “PASS,” and AD_TUMOR >= 5. The filtered variants were used for mutational signature analysis, using SigProfilerMatrixGenerator v1.2 and SigProfilerExtractor v1.1.4 from Alexandrov Laboratory at University of California at San Diego (UCSD) were used.

### In vitro drug treatment (GEMINI assay)

DNMT1i treatment of the BCP-ALL cell line (T259) was performed using a Genotoxic Mutational Signature Identified After Clonal Expansion In Vitro (GEMINI) assay.^17^ Briefly, T259 cells were single-cell sorted into 96-well plates. A single well was expanded and treated in 25 mL flask with either nonazanucleoside DNMT1i, GSK-3685032 (GSK), ATC, or both GSK and ATC for 12 days, refreshing IMDM complete media and DNMT1i every 3 days. Treated cells were single-cell sorted and expanded in 96-well plates. Three clones were randomly selected and expanded for WES.

## Results

### In vivo ATC treatment of RAG-1 KO mice leads to B-lineage ALL

**Figure 1.**
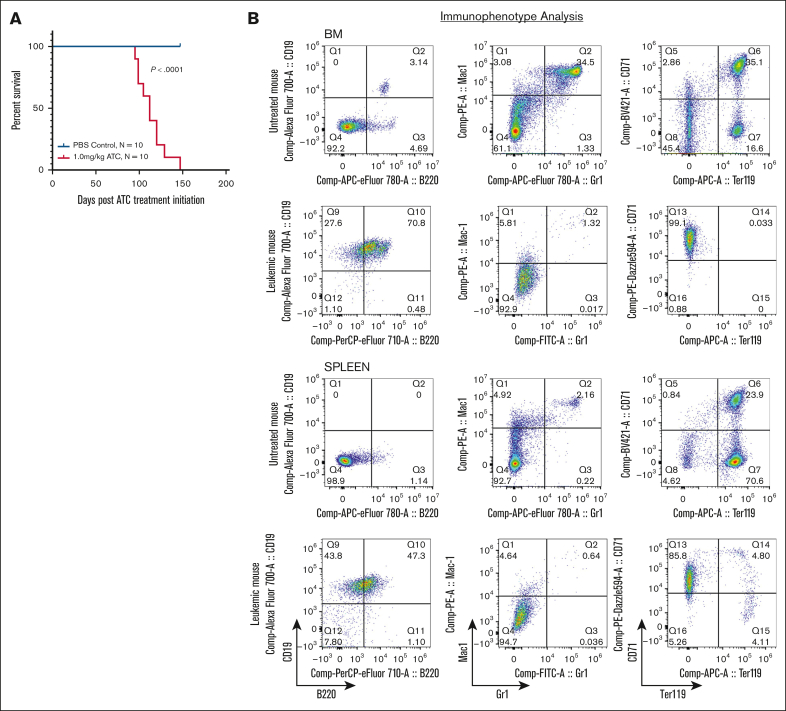
**ATC treatment leads to B-lineage ALL in RAG-1 KO mice.** (A) Survival of RAG-1 KO mice treated with ATC or phosphate-buffered saline (PBS). Log-rank (Mantel-Cox) *P* < .0001 for ATC-treated mice vs PBS treated mice. Days represent time postinitiation of ATC treatment. (B) Flow cytometry of BM and spleen from an untreated RAG-1 KO mouse and a moribund ATC-treated RAG-1 KO mouse (8664) stained for CD19, B220, Mac1, Gr1, CD71, and Ter119.

*Rag-1*^–/–^ (RAG-1 KO) mice produce no mature T or B cells due to a complete lack of variable diversity joining (VDJ) recombination resulting in a developmental arrest of T and B lymphocytes.^18^ RAG-1 KO mice were treated with repeat cycles of ATC (1 mg/kg) or phosphate-buffered saline as a control. One cycle consisted of once-daily intraperitoneal injections 5 days per week for 2 weeks, followed by 1-week rest. Cycles were repeated until the mouse showed signs of morbidity requiring humane euthanasia. Mice treated with ATC became ill as early as 100 days postinitial exposure, with a median survival of 112 days. In contrast, none of the phosphate-buffered saline treated RAG-1 KO mice showed signs of illness during the study period (Figure 1A; supplemental Table 1).

Euthanized mice showed nonspecific signs of illness consistent with leukemia, such as weakness, lethargy, and hunched posture. Necropsy revealed hepatomegaly and splenomegaly, and complete blood count showed increased lymphocyte count, anemia, and thrombocytopenia (supplemental Figure 1A-C). Surprisingly, flow cytometry of cells from BM, spleen and thymus showed a uniform CD19^+^B220^+^ population, suggesting a B-lineage leukemia (Figure 1B). Additionally, we observed a decrease in myeloid (Mac1^+^Gr1^+^) and erythroid (CD71^+^Ter119^+^) cells suggesting loss of normal hematopoiesis (Figure 1B). The malignant CD19^+^B220^+^ population was observed in all evaluable ATC-treated mice (3 mice were found dead and unable to be further evaluated) (supplemental Figure 1D). Supplemental Table 1 summarizes the clinical and laboratory findings from ATC-treated RAG-1 KO mice.

In addition, we observed 3 cases of thymocyte expansion, likely due to precursor T-cell lymphoblastic lymphoma (pre–T-LBL) in thymic tissue of mice 8656, and 8660, all with concurrent B-lineage ALL in BM and spleen. These pre–T-LBL showed >20% CD8^+^ cells (supplemental Figure 1E), which likely reflects an expansion of immature CD8 single-positive thymocytes cells characterized by CD8 expression but absence of T-cell receptor β.^19^ Taken together, these findings demonstrate that RAG-1 KO mice treated with ATC develop B-lineage ALL, with a subset exhibiting concurrent T-lineage malignancy.

### Identification of BCPs as a potential target for ATC-induced leukemic transformation

**Figure 2.**
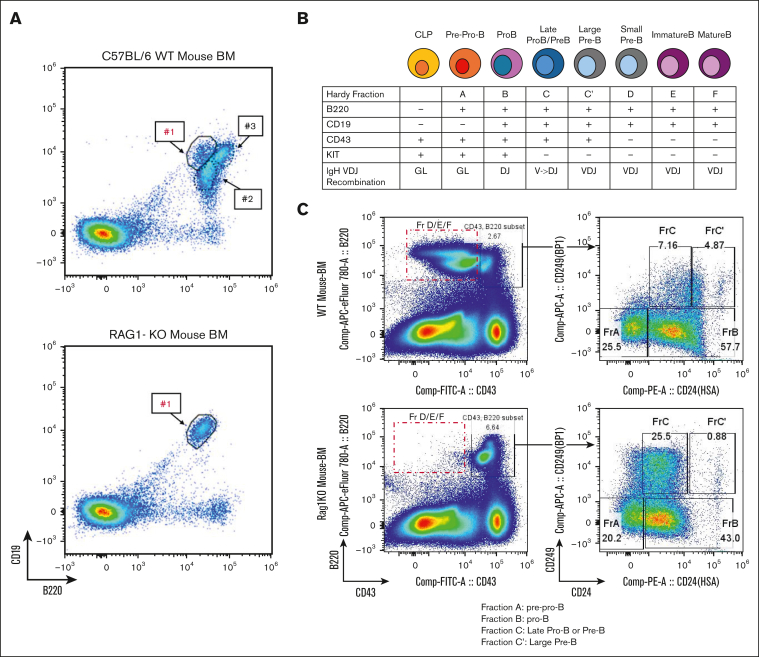
**BCPs in RAG-1 KO BM are potential targets for ATC-induced leukemic transformation.** (A) Flow cytometry analysis of untreated C57BL/6 and untreated RAG-1 KO BM; #1, #2, and #3 represent 3 discrete populations of CD19^+^B220^+^ cells. (B) Schematic of BM B-cell differentiation based on surface markers and IgH VDJ recombination. (C) Hardy fraction staining of untreated RAG-1 KO and WT mouse. Red box indicates B220^+^CD43^–^ progenitors missing in RAG-1 KO mouse. RAG-1 KO mouse shows accumulation of Hardy fraction C and decreased C' compared WT BM. CLP, common lymphocyte progenitor; GL, germ line; IgH, immunoglobulin heavy chain.

The development of B-cell (and to a lesser extent, T-cell) malignancies in RAG-1 KO mice was unexpected, as these mice lack circulating B or T cells. We hypothesized that the malignancy may originated from committed B (or T) cell precursors that had not yet undergone immunoglobulin or T-cell receptor gene rearrangement. Supporting this, flow cytometry of BM from untreated RAG-1 KO mice identified a single CD19^+^B220^+^ population (Figure 2A), whereas wild type (WT) BM demonstrated 3 distinct CD19^+^B220^+^ populations, only one of which was detected in RAG-1 KO BM (population 1 in Figure 2A).^20^

Normal B-cell development in BM can be fractionated into Hardy fractions, with Hardy fraction A being the least and fraction F the most mature BCPs (Figure 2B).^21^^,^^22^ RAG-1 KO BM B cells are able to differentiate to fractions A, B, and C, but not fractions C', D, E, or F (Figure 2C), suggesting that these BCPs are blocked at Hardy fraction C, likely due to the absence of VDJ recombination. We conclude that RAG-1 KO mice treated with ATC invariably develop BCP-ALL, and that Hardy fraction B/C BCPs are likely targets for ATC-induced leukemic transformation.

ATC’s oncogenic effect depends on deoxycytidine kinase (Dck), which phosphorylates ATC, allowing incorporation into DNA.^17^ Of note, Hardy fraction B/C cells express the highest levels of *Dck* amongst all B-cell populations (supplemental Figure 2A). Therefore, accumulation of Hardy fraction B/C cells (due to the RAG-1 KO differentiation block) would make these Hardy fraction B/C precursors ideal targets for ATC incorporation into DNA, resulting in ATC-induced mutagenesis, and culminating in leukemic transformation of BCPs. As shown in supplemental Figure 2B, there was abundant *Dck* expression in CD19^+^B220^+^ cells purified from nonleukemic RAG-1 KO BM. Additionally, we observed high expression of *Dck* for immature single positive (SP) thymocytes (supplemental Figure 2C) supporting the hypothesis that immature CD8 single positive (SP) thymocytes cells could be the normal counterpart for the concurrent pre–T-LBL observed in the thymic tissue of mice 8656, 8659, and 8660.

### Gene expression profile supports the diagnosis of BCP-ALL

**Figure 3.**
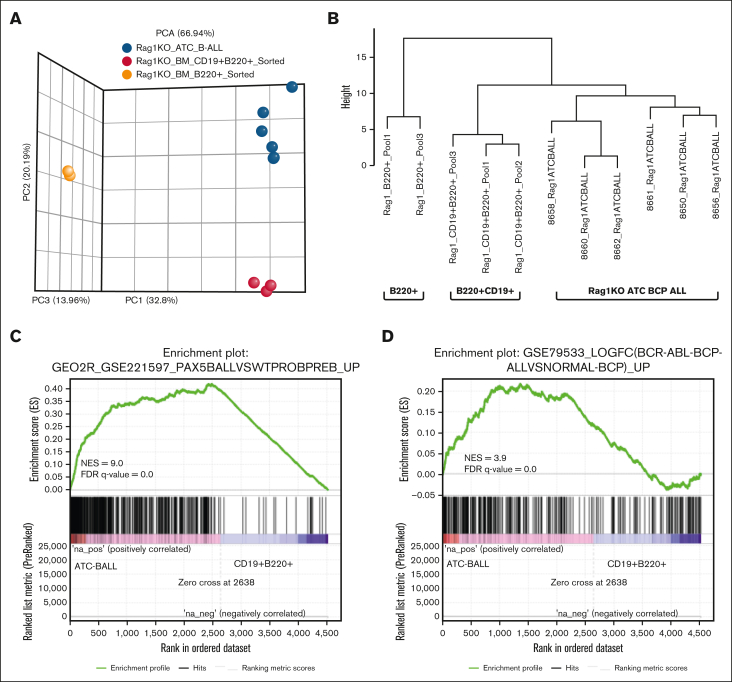
**Gene expression analysis highlights enrichment of both murine and human BCP-ALL signatures in RAG-1 KO ATC CD19^+^ B220^+^ B-lineage ALL.** (A) Principal component analysis plot generated from DeSeq2 normalized RNA sequencing data. Samples include 6 RAG-1 KO ATC B-lineage leukemias, 3 untreated RAG-1 KO CD19^+^B220^+^ sorted BM cells and 2 RAG-1 KO B220^+^ sorted BM cells. (B) Hierarchical clustering for selected genes associated with B-cell development. (C) GSEA comparing a murine BCP-ALL gene set (GSE221597) and differentially expressed genes from RAG-1 KO BCP-ALL samples (n = 6) vs CD19^+^B220^+^ sorted BM cells (n = 3). (D) GSEA comparing human BCP-ALL gene set (GSE79533) and differentially expressed genes from RAG-1 KO BCP-ALL vs CD19^+^B220^+^ sorted BM. FDR, false discover rate; NES, normalized enrichment score.

Gene expression profiling was used to further validate BCP-ALL diagnosis. Controls were flow-sorted CD19^+^B220^+^ and B220^+^ cells from clinically healthy, untreated RAG-1 KO BM (supplemental Table 2). Principal component analysis showed that samples clustered according to expected phenotypic groups (Figure 3A). Hierarchical clustering based on genes important for B-cell differentiation^23^ (supplemental Table 3) demonstrated that the CD19^+^B220^+^ BCP-ALL were more similar to nonmalignant CD19^+^B220^+^ double-positive cells than nonmalignant B220^+^ SP cells (Figure 3B).

GSEA comparing RAG-1 KO BCP-ALL to murine BCP-ALL (GSE221597) revealed enrichment of genes expressed in ATC-induced leukemias (Figure 3C). Similar analysis of genes upregulated in human BCP-ALL (GSE79533) showed enrichment of the ATC-induced BCP-ALL in several human BCP-ALL gene sets (Figure 3D; supplemental Figure 3A).

GSEA analysis using annotated gene sets from Molecular Signature Database showed enrichment of RAG-1 KO BCP-ALL in gene sets associated with extracellular matrix organization, integrin cell surface interactions, collagen biosynthesis and cell adhesion molecules (supplemental Figure 3B). Leading edge analysis performed using these gene sets highlighted collagen gene family genes, integrins and other adhesion genes, which were consistently overexpressed in RAG-1 KO BCP-ALL samples (supplemental Figure 3C). Previous studies have identified adhesive interaction of human BCP-ALL cells with BM stromal cells and implicated the role of molecules such as collagen, integrins and other extracellular matrix proteins in these interactions.^24^^,^^25^ Taken together, these findings reinforce the diagnosis of BCP-ALL in ATC-treated RAG-1 KO mice and its similarity of human disease.

### Mutational burden in ATC-induced BCP-ALL

**Figure 4.**
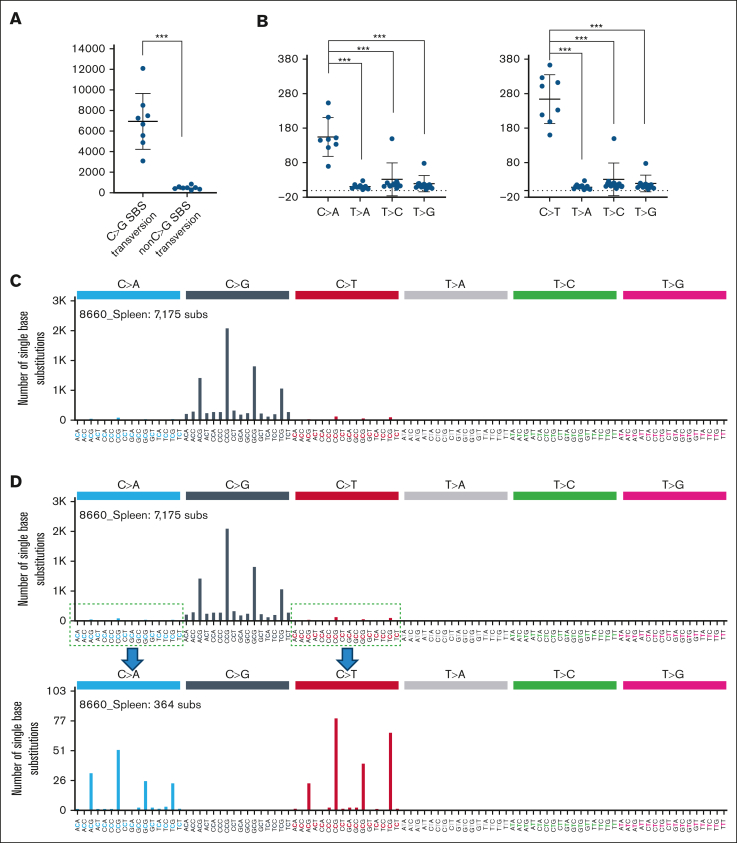
**BCP-ALL developing after ATC exposure shows a dramatic increase in C>G transversions.** (A) Absolute number of C>G and non-C>G transversions identified for RAG-1 KO ATC B-lineage leukemia samples (n = 8) using WES. (B) Absolute number of C>A (left plot) and C>T (right plot) SBS mutations compared to remaining SBS mutations (excluding C>G transversions) identified from WES data. ∗∗∗*P* = .0002. (C) Representative SBS plot of RAG-1 KO BCP-ALL from mouse 8660. Note most C>G transversions are in a 5'-NCG-3' context. (D) SBS plot of RAG-1 KO BCP-ALL from mouse 8660 following computational removal of C>G mutations and re-scaling mutation count; this allows visualization of non-C>G mutations. C>A and C>T mutations are also in a 5'-NCG-3' context.

WES of the ATC-induced BCP-ALL revealed a dramatic increase in C>G mutations (an average of 6948 mutations per leukemic sample) compared to non-C>G mutations (an average of 496 mutations per leukemic sample) (Figure 4A; supplemental Table 4). C>A and C>T mutations were also increased in ATC-induced BCP-ALL, albeit to a far lesser extent than C>G mutations (Figure 4B). SigProfiler analysis demonstrated that C>G transversions occurred predominantly in a 5'-NCG-3' context, (Figure 4C). This single base substitutions (SBS) signature was highly reproducible across all RAG-1 KO leukemia samples, including 7 BCP-ALL and 1 pre–T-LBL (8660_Thymus) (supplemental Figure 4A), with a consistent preference for the following order: CCG > GCG > ACG > TCG (Figure 4C; supplemental Figure 4A). We compared the ATC-induced mutational signature with known mutational signatures using SigProfiler Assignment tool, and found similarity with SBS98 (https://cancer.sanger.ac.uk/signatures/sbs/) (supplemental Figure 4B-C). The etiology of SBS98 is unknown, but suggested to be similar to SBS87, which has been linked to thiopurine treatment.^26^, ^27^, ^28^ To focus on C>T and C>A mutational signatures, we computationally removed C>G mutations and determined the mutational signature of the modified dataset (Figure 4D; supplemental Figure 5A). SigProfiler Assignment analysis of this modified dataset identified a prominent SBS87 as well as SBS1, SBS10b and SBS49 (supplemental Figure 5B-C).

Given that C>T mutations also arise by spontaneous deamination of methylated cytosine,^29^^,^^30^ leading to COSMIC SBS1 as seen above, we compared the 5'-NCG-3' trinucleotide context for C>T mutations in the RAG-1 KO leukemia to SBS1. The ATC-induced C>T signature was different than SBS1, with the preference for purines and pyrimidines at the “N” position being reversed (supplemental Figure 5C), suggesting that the mechanism for ATC-induced C>T mutations may be distinct from SBS1.

To determine if there was a preference for nucleotides beyond the trinucleotide core in generation of C>G transversions, we expanded the nucleotide sequence flanking the C>G mutations to a 5-nucleotide context. We did not find a difference between the sequence of nucleotides flanking recurrent (those occurring in >1 leukemia sample) and nonrecurrent C>G mutations at CpG sites (supplemental Figure 6A), suggesting that there was no influence from the additional nucleotides. Similar analysis focused on the C>G mutations at non-CpG sites also showed no evidence that flanking nucleotides influenced mutation likelihood (supplemental Figure 6A-B).

C>G transversions in a CpG context were 2.6 times more common than C>G in a non-CpG context (supplemental Figure 6C), even though C in a CpG context is markedly underrepresented in the mammalian genome (<1% of all possible dinucleotides).^31^ This preference for CpG sites was exaggerated for C>A and C>T mutations (supplemental Figure 6C). Taken together, these findings suggest that cytosine mutations at CpG dinucleotide may involve a different mechanism than cytosine mutations at non-CpG dinucleotide.

### Mutations in genes associated with human B-ALL suggest a mechanism for leukemogenesis

**Figure 5.**
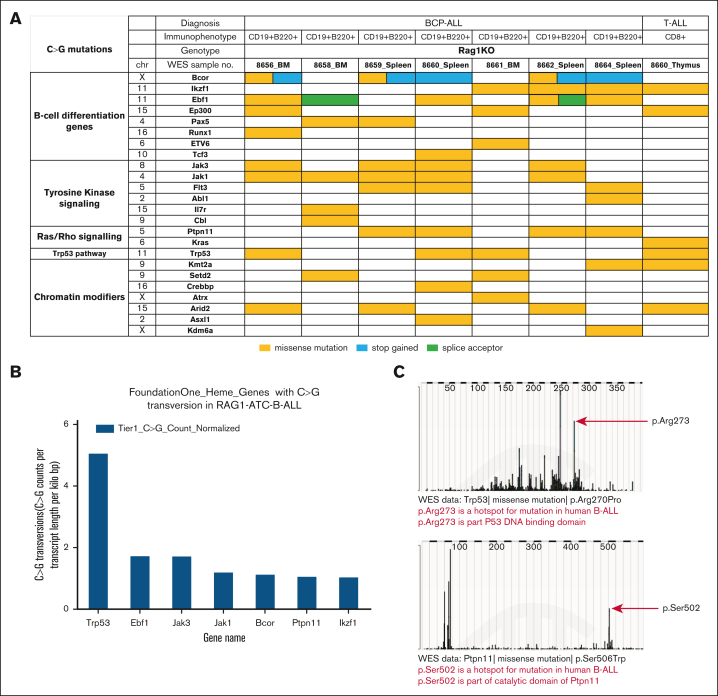
**ATC treatment of RAG-1 KO mice leads to acquired mutations in genes important for cancer.** (A) Acquired C>G mutations involving genes important for cancer in ATC-treated RAG-1 KO leukemia. (B) Most common Tier 1 C>G transversions (identified from WES data of RAG-1 KO ATC B-lineage leukemias), normalized for gene length (see “Materials and methods”). (C) C>G mutation at mutational hot spots in human B-ALL. Murine *Trp53* R270 is equivalent to human *TP53* R273 and murine *Ptpn11* S506 is equivalent to human *PTPN11* S502.

We examined the WES data to identify acquired mutations in ATC-treated RAG-1 KO leukemia samples. To identify mutations that were relevant for human hematologic malignancy, we compared C>G transversions involving Tier 1 (HIGH or MODERATE impact) protein coding variants with variant allele frequencies >0.2 to genes included in a commercial hematologic malignancy gene panel (432 genes; FoundationOne Heme) (Figure 5A; supplemental Figure 7A-B). Most of the BCP-ALL samples had mutations in more than one relevant pathway, such as B-cell differentiation, proliferation signaling, and epigenetic modifiers. Recurrent variants were found in genes known to be relevant for human BCP-ALL including *Trp53*, *Ebf1*, *Jak3*, *Ptpn11*, *and Ikzf1* (Figure 5B); a number of these mutations occurred at the murine homologue of known human “hot spot” amino acid residues that are associated with malignant transformation, including *Pax5* P80R, *Ptpn11* S506W, and *Trp53* R270P (Figure 5C; supplemental Table 4). These data support the hypothesis that ATC-induced C>G transversions at important oncogenes collaborate and are selected for a fitness advantage in vivo, resulting in malignant transformation.

B-cell malignancies are typically characterized by clonal *IGH* gene rearrangement.^32^ However, RAG-1 KO mice are unable to undergo VDJ recombination due to absence of a functional RAG-1 protein. To verify that RAG-1 KO BCP-ALL were unable to undergo clonal VDJ recombination we examined .bam files from WES. As a positive control for clonal VDJ rearrangement, we used T259 cells, a murine BCP-ALL cell line^33^ that has a clonal *Igh* rearrangement involving *Ighj4* and *Ighv2-2* (supplemental Figure 8A). We did not find any evidence for VDJ rearrangements in the RAG-1 KO BCP-ALL samples, which all had germ line *Igh* J-region configuration, identical to nonmalignant tail DNA (supplemental Figure 8B).

### Methylation status of cytosines targeted for mutation

**Figure 6.**
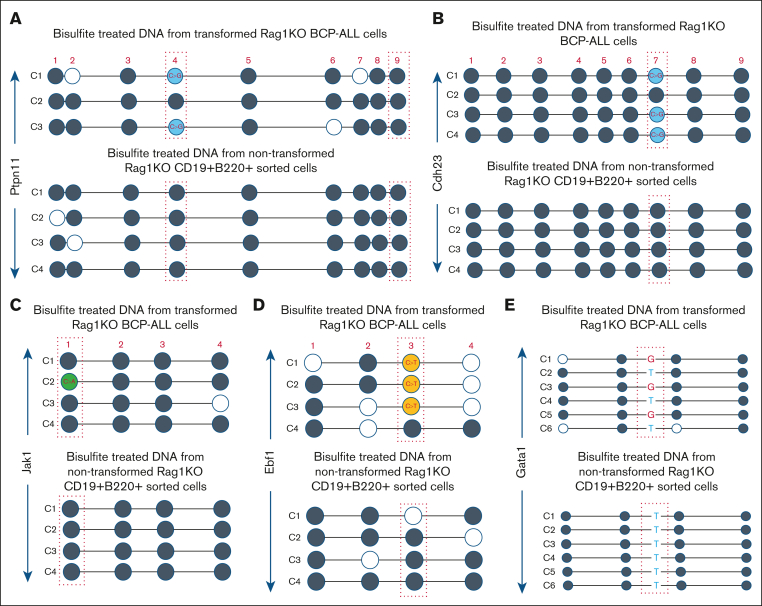
**CpG dinucleotides subject to ATC-induced C>G transversions are highly methylated.** (A) Methylation status of C>G mutated CpG for *Ptpn11*. C1-C3 represent individual plasmid subclones from polymerase chain reaction amplification of bisulfite treated RAG-1 KO leukemic DNA (top), and C1-C4 represent subclones from bisulfite-treated RAG-1 KO nonleukemic BCP DNA (bottom). Individual CpG are numbered 1-9. Dotted red boxes indicate CpG at known sites of C>G transversion in this study. Dark gray circles indicate methylated cytosines, white circles indicate unmethylated cytosines, and blue circles indicate C>G transversions. (B) Methylation status of C>G mutated CpG dinucleotides at *Cdh23*. (C) Methylation status of C>A mutation at CpG dinucleotides within *Jak1*. Green circle indicates a C>A mutation known to be present. (D) Methylation status of C>T mutation at CpG dinucleotide within *Ebf1*. Orange circles indicate a C>T mutation known to be present. (E) Methylation status of C>G transversion at non-CpG dinucleotides within *Gata1*. G in red represents C>G mutation at a non-CpG dinucleotide which was known to be present in this sample based on WES. “T” in blue represents bisulfite conversion of nonmethylated C.

Most C>G (as well as C>T and C>A) mutations occurred at CpG dinucleotides, suggesting that cytosine methylation status may be related to the ATC-induced mutations, given that almost all mammalian DNA methylation takes place on cytosines in a CpG context.^34^ We used bisulfite specific polymerase chain reaction and sequencing to assess the methylation status of nucleotides that were subject to C>G transversions in the ATC-induced BCP-ALL.^35^ We assessed the methylation status in both leukemic samples as well as nonmalignant BCPs (the cell type and differentiation stage most likely to have become transformed). Because the mutation data could be skewed by in vivo selection of specific oncogenic mutations, we examined genes known to be involved in human BCP-ALL (*Ptpn11*, *Bcor*, and *Trp53*) as well as genes that are not known to be involved in BCP-ALL (*Cdh23*, *Pcdha12*, and *Adgrb3*). For all genes examined, the CpG sites with known C>G mutations (found in the WES data) were methylated in leukemia samples and nonleukemic BCPs (Figure 6A-B; supplemental Figure 9A-B). A similar methylation analysis demonstrated that C>T and C>A mutations at CpG dinucleotides were heavily methylated; this was true for both leukemic cells and their non leukemic counterparts (Figure 6C-D). As expected, no C>G, C>A, or C>T mutations were identified in any bisulfite treated DNA subclone from the untreated, nonleukemic BCPs. This data demonstrates that methylated cytosines in a CpG context were preferential targets for ATC-induced C>G transversions.

Although most C>G transversions occurred in a CpG context, a significant minority of C>G transversions occurred at non-CpG cytosines. Therefore, we examined the methylation status in regions of known C>G mutations at non-CpG sites; as anticipated, these cytosines were universally un-methylated (Figure 6E). This suggested to us that ATC-induced SBS at non-CpG dinucleotides may involve a different mechanism than SBS at CpG dinucleotides.

**Figure 7.**
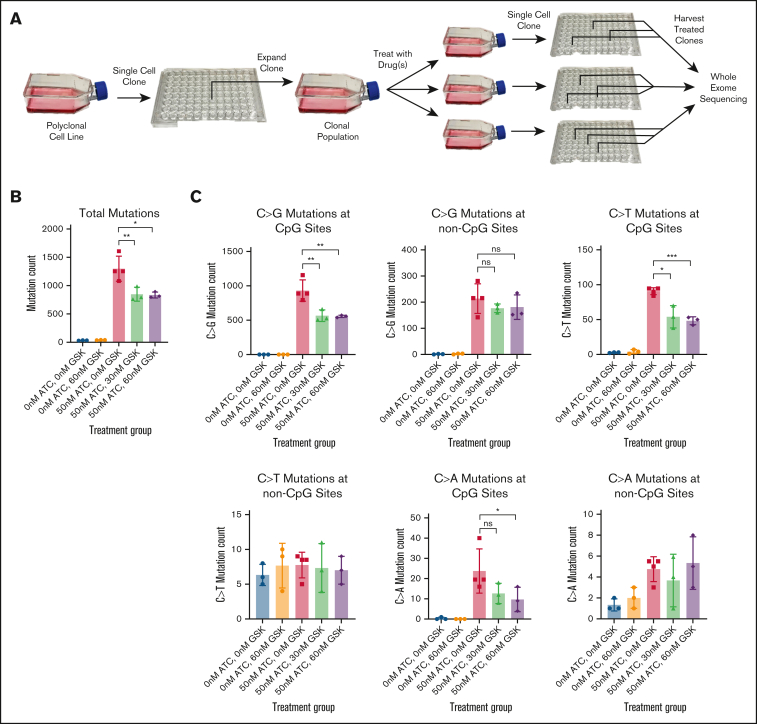
**Mutations induced at CpG sites require DNMT1, whereas mutations at non-CpG sites do not.** (A) Schematic of the GEMINI Assay used for in-vitro drug treatment and subsequent WES. (B) Total mutations induced in each treatment group. Unequal variances *t* test (1 tailed). (C) C>G, C>T, and C>A mutations induced at CpG sites and non-CpG sites. Unequal variances *t* test (1-tailed); ∗, *P* < .05; ∗∗, *P* < .01; ∗∗∗, *P* < .001; ns, not significant.

A previous study that investigated a potential role for DNMT1 protein-DNA adducts in the generation of C>G transversions following treatment with a related azanucleoside (5-azacytidine) predicted that DNMT1 was directly involved in the generation of C>G mutations in a CpG context.^36^ To determine if inhibition of DNMT1 would decrease C>G mutations induced by ATC, we used an in vitro GEMINI (Genotoxic Mutational Signature Identified After Clonal Expansion In Vitro) assay.^17^ In this assay, flasks containing clonally identical populations of cells are treated in-vitro with a mutagen for a specified period of time, single cell cloned and then analyzed for acquired mutations using whole genome or WES (Figure 7A). In this experiment, a murine BCP-ALL cell line, T259,^33^ was treated with a nonazanucleoside DNMT1i, GSK-3685032 (GSK),^37^ ATC, or both GSK and ATC. Individual single cell clones from each treatment were randomly selected and analyzed by WES (supplemental Table 5). As shown in Figure 7B, treatment with 60 nM GSK alone for 12 days did not induce mutations in the treated cells, whereas treatment with 50 nM ATC alone for 12 days led to an average of 1299 total mutations. This ATC-induced mutagenesis is expected based on prior GEMINI assays using ATC.^17^ Notably, treatment with both 50 nM ATC and GSK (either 30 or 60 nM) led to a mean of 848 and 834 mutations, a significant decrease compared to ATC alone (Figure 7B). This reduction in induced mutations by DNMT1 inhibition supports the hypothesis that DNMT1 enzymatic activity was involved in the ATC-induced generation of mutations in these cells.

We then divided the induced mutations based on location at CpG sites or non-CpG sites (Figure 7C). 50 nM ATC treatment induced a mean of 931 C>G mutations at CpG sites and 214 C>G mutations at non-CpG sites. 50-nM ATC also induced a mean of 91 C>T and 24 C>A mutations at CpG sites, but almost no C>T or C>A mutations at non-CpG sites (Figure 7C). These findings suggest that C>G mutations induced at non-CpG sites may occur through a different mechanism than C>G, C>T, or C>A mutations at CpG sites, as C>G mutations are the only cytosine mutations that occur in a non-CpG context (i.e., no C>T or C>A mutations in a non-CpG context). Notably, concurrent GSK and ATC treatment significantly reduced C>G and C>T mutations located at CpG sites, but not at non-CpG sites (Figure 7C). This finding supports the hypothesis that DNMT1 enzymatic activity is important for the ATC-mediated induction of C>G, C>T, and C>A mutations at CpG sites, but not for C>G mutations induced at non-CpG sites.

## Discussion

In this study, we have shown that exposure of RAG-1 KO mice to the cytidine analog ATC results in malignant transformation of BM BCPs. ATC exposure produces a unique mutational signature highlighted by increased C>G transversions in a specific 5'-NCG-3' context. Bisulfite assays demonstrated that cytosine residues in a CpG context targeted for mutation were heavily methylated, and inhibition of DNMT1 reduced C>G mutations in a CpG context, both suggesting a role for *Dnmt1* in mutations produced by ATC. Several genes relevant for human BCP-ALL were mutated in the ATC exposed samples, thus linking a specific mutagen to a specific mutational signature and malignant transformation.

The gene expression profile of B lineage leukemias that developed in RAG-1 KO mice following ATC exposure was similar to that seen with both murine and human BCP-ALL. Additionally, we identified upregulation of pathways involving extracellular matrix organization, which have been reported to promote progression of human BCP-ALL by facilitating leukemia cell survival, growth, and invasion.^24^^,^^25^^,^^38^ Although speculative, it is possible that the hind leg paralysis observed in several leukemic mice (supplemental Table 1) was due to spinal cord involvement with leukemic cells expressing adhesion molecules.

Development of BCP-ALL in RAG-1 KO mice was initially unexpected, given that RAG-1 KO mice do not produce mature B or T-cells, due to the lack of VDJ recombination. However, further study revealed that RAG-1 KO mice do generate BCPs that are developmentally blocked at Hardy fraction C, providing targets for leukemic transformation. The oncogenic ability of ATC is highly dependent on DCK enzymatic activity, which phosphorylates ATC, allowing for its incorporation into DNA.^17^ Of note, Hardy fraction B/C cells express the highest levels of *Dck* amongst all B-cell populations. Therefore, accumulation of Hardy fraction B/C cells (due to the RAG-1 KO differentiation block), along with the high expression of *Dck*, makes these Hardy fraction B/C precursors ideal targets for ATC incorporation resulting in mutagenesis.

Prior studies have highlighted networks of cooperative mutations that lead to BCP-ALL.^39^ Conceptually, an initiating lesion perturbs genes associated with epigenome or self-renewal, and subsequent lesions target pathways controlling differentiation and proliferation.^39^ We identified ATC-induced mutations involving combinations of genes that were selected for in vivo. These included mutations in *Kmt2a*, *Setd2*, or *Crebbp* (initiation lesions), *Pax5*, *Bcor*, or *Ebf1* (impaired differentiation) and *Jak1*, *Jak3*, *Kras*, or *Ptpn11* (abnormal proliferation). As examples of cooperative lesions, mouse 8660 had mutations in *Crebbp*, *Bcor*, *Ebf1*, and *Jak1/3*, and mouse 8664 had mutations in *Kdm6a*, *Bcor*, *Ebf1*, *Flt3*, and *Ptpn11*.

The RAG-1 KO BCP-ALL is similar to human BCP-ALL in terms of immunophenotype, gene expression profile and acquired somatic mutations. We speculate that ATC mutagenesis could be used to potentiate incompletely penetrant models, such as those that expressing an ETV6::RUNX1 (TEL::AML1) fusion,^40^^,^^41^ in a fashion analogous to retroviral insertional mutagenesis.^42^ In theory, mutations induced by ATC would collaborate with the fusion (eg, ETV6::RUNX1), and a malignant clone with a fitness advantage would emerge. Instead of a retroviral “tag” ATC-induced mutations could be identified as C>G (or C>T, C> A) variants in a CpG context. Moreover, the ATC-induced mutagenic system potentially could be adapted to study specific subsets of BCPs and preleukemic cells, as well as the leukemogenic potential and functional consequences of particular mutations in these B-cell subsets.

We identified 3 types of SBS mutation signatures in ATC-treated RAG-1 KO mice. The first and most abundant was a C>G transversion in a CpG context. The second comprised C>A and C>T substitutions, also in a CpG context; these mutations have similarity to SBS98 and SBS87 signatures, both linked to treatment with the nucleoside analog 6-thioguanine. The third signature was characterized by C>G transversions in a non-CpG context. We propose that the mutations occurring in a CpG context involve a mechanism requiring DNMT1 activity. This model is based on prior speculation that DNMT1 bound to an azanucleoside forms a protein-DNA adduct, which is resolved by opening of the azanucleoside ring into a structure capable of abnormal hydrogen bonding^36^ (supplemental Figure 10A). Following mispairing with a normal cytosine, the next round of DNA replication leads to a C>G transversion. Less common C>T or C>A mutations in a CpG context would occur in a similar fashion, either due to mispairing with T or A, or repair of an abasic site following removal of the ring open azacytosine remnant. To explain the increased C>G mutations in a non-CpG context, we speculate that less frequent spontaneous hydrolysis of the azacytidine ring results in ring-opening at non-CpG dinucleotides, followed by mispairing with a normal cytosine residue (supplemental Figure 10B). This mechanism is independent of DNMT1 and does not lead to increased C>T or C>A mutations in a non-CpG context, consistent with our experimental findings.

We have shown that ATC has a strong mutagenic effect in vivo, most prominently leading to increased C>G mutations. Mutations involving genes critical for self-renewal, B-cell differentiation, and control of proliferation collaborate and lead to malignant transformation of BCPs in vivo. We present a model in which CpG methylation and Dnmt1 activity are important mediators of these C>G transversions in a CpG context, and we speculate that Dnmt1 activity is not required for ATC-induced mutations that occur in a non-CpG context. These findings suggest that therapy-related malignancies arising after cytidine analog therapy warrant careful examination of their mutational signatures.

Conflict-of-interest disclosure: The authors declare no competing financial interests.

## Supplementary Material

Supplemental Figures, Methods, and References
