# Supplementary material for: Azanucleoside treatment leads to B-cell precursor acute lymphoblastic leukemia
Source: Blood Neoplasia. 2025 Aug 19;2(4):100161. doi: 10.1016/j.bneo.2025.100161 (PMC12555782; doi:10.1016/j.bneo.2025.100161)
Supplement: Supplemental Figures, Methods, and References [file BNEO_NEO-2025-000735-mmc1.pdf]

Supplementary Figure S1

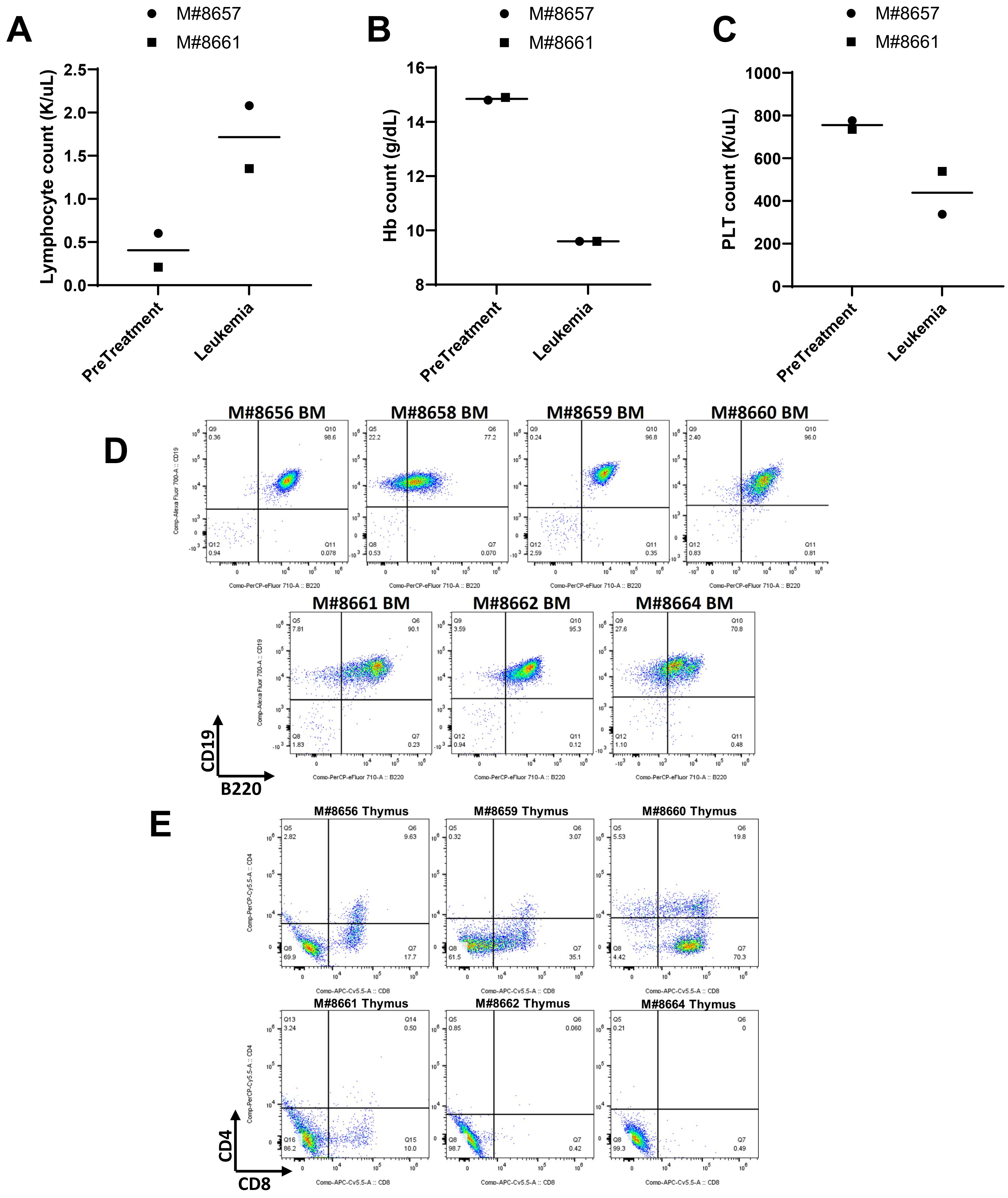

Supplementary Figure S1. Characterization of leukemic RAG-1 KO mice.

A-C. Lymphocyte, hemoglobin, and platelet count before beginning experiment and at the time of euthanasia. **D.** Flow cytometry showing CD19 and B220 staining of BM from leukemic mice. **E**. CD4 and CD8 staining of thymic tissue from leukemic mice. Mice #8656, #8659 and #8660 show >20% CD8+, suggesting concurrent aberrant T lineage expansion in addition to B lineage ALL in BM.

Supplementary Figure S2

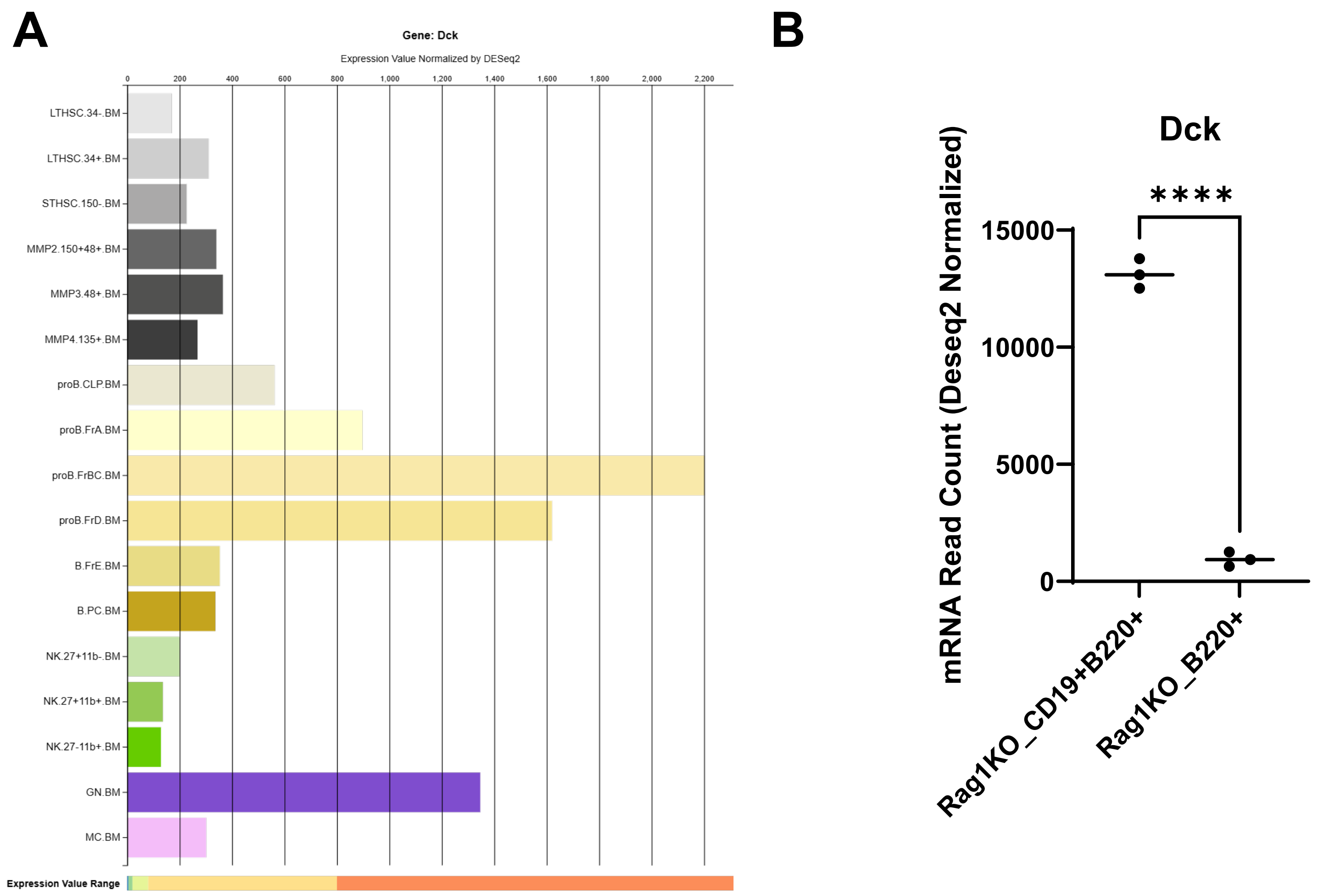

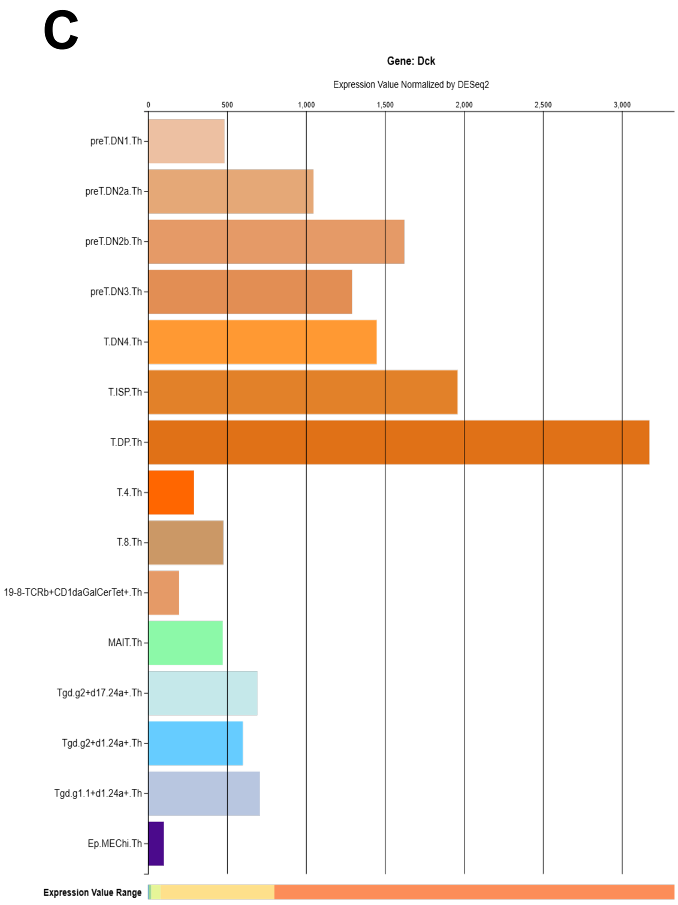

Supplementary Figure S2. Murine *Dck* expression in T and cell subsets.
A. Expression of *Dck* in stem, B cell subsets and myeloid lineage cells in BM. Data from Immunological Genome Project (ImmGen)(https://rstats.immgen.org/Skyline/skyline.html), Gene Skyline (ULI RNA-seq data). **B.** RNA-Seq (Deseq2 normalized) read count for untreated RAG-1 KO CD19+B220+, and untreated RAG-1 KO B220+ single positive BM cells. RAG-1 KO CD19+B220+ cells express significantly more Dck than RAG-1 KO B220+ cells. **** Unpaired t test *P*<0.0001. **C.** *Dck* expression in thymic subsets; data from ImmGen.

Supplementary Figure S3

A

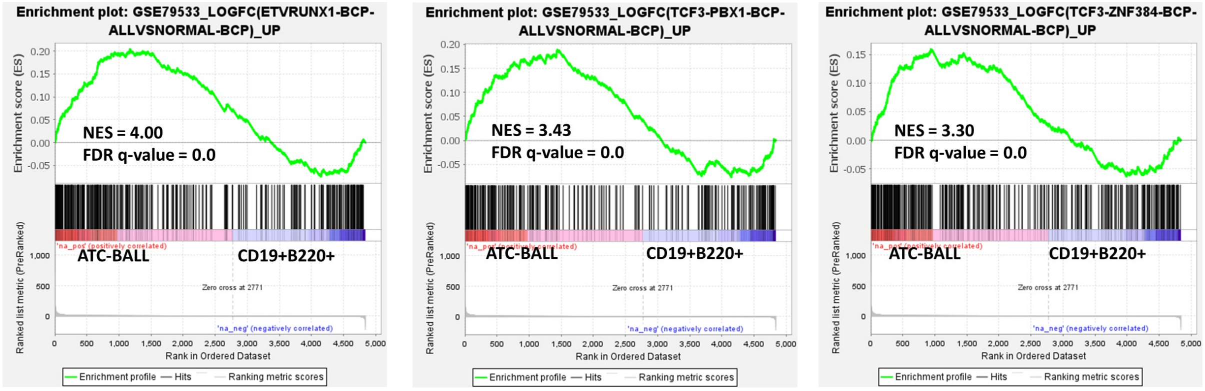

B

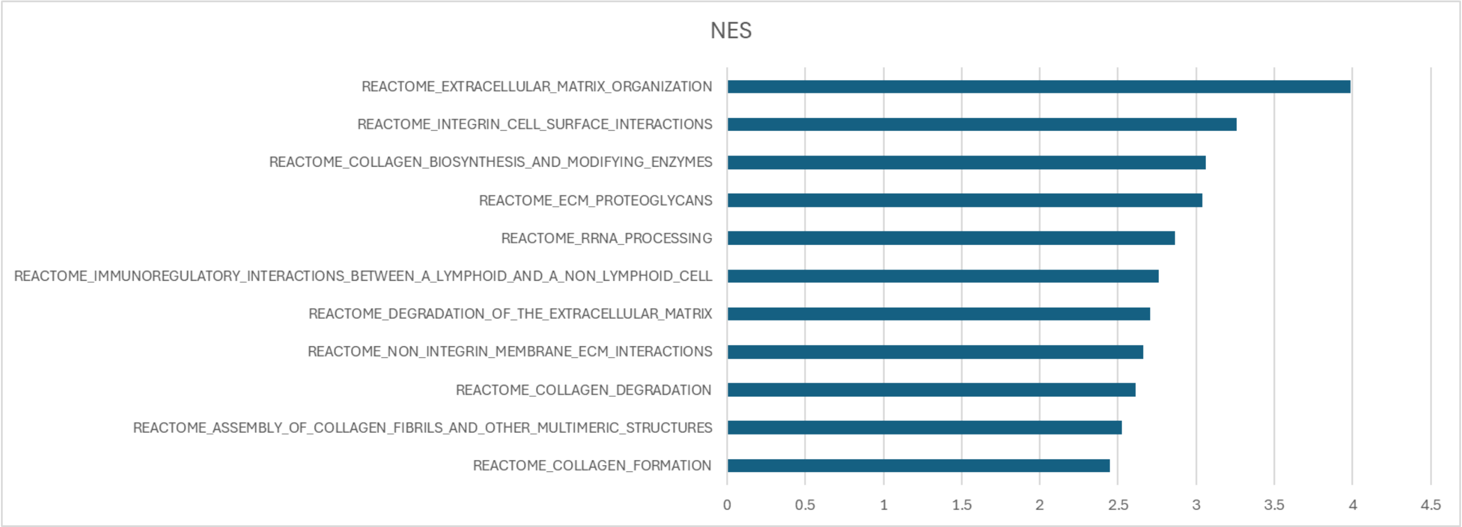

C

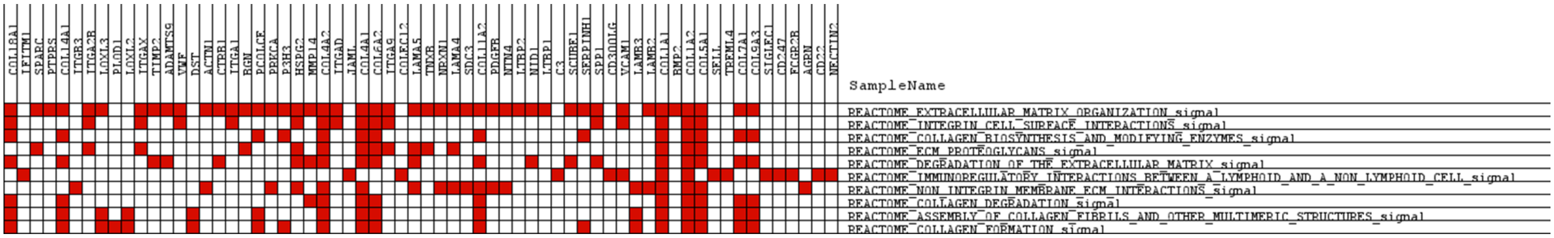

Supplementary Figure S3. GSEA analysis showing enrichment of human BCP-ALL signature and pathway associated with extracellular matrix and integrins in RAG-1 KO ATC CD19+ B220+ B-lineage ALL .

A. GSEA using human BCP ALL gene sets (GSE79533) compared to differentially expressed genes from RAG-1 KO BCP ALL vs CD19+B220+ sorted BM**. B.** GSEA using curated gene sets from c2.cp.reactome.v2024 (Molecular Signature Database (MSigDB)) showing most positive normalized enrichment score with p-value = 0 for RAG-1 KO BCP ALL vs RAG-1 KO CD19+B220+ cells. **C**. Leading edge analysis using gene sets from c2.cp.reactome.v2024 (Molecular Signature Database (MSigDB)) in which RAG-1 KO BCP ALL genes were positively enriched.

Supplementary Figure S4

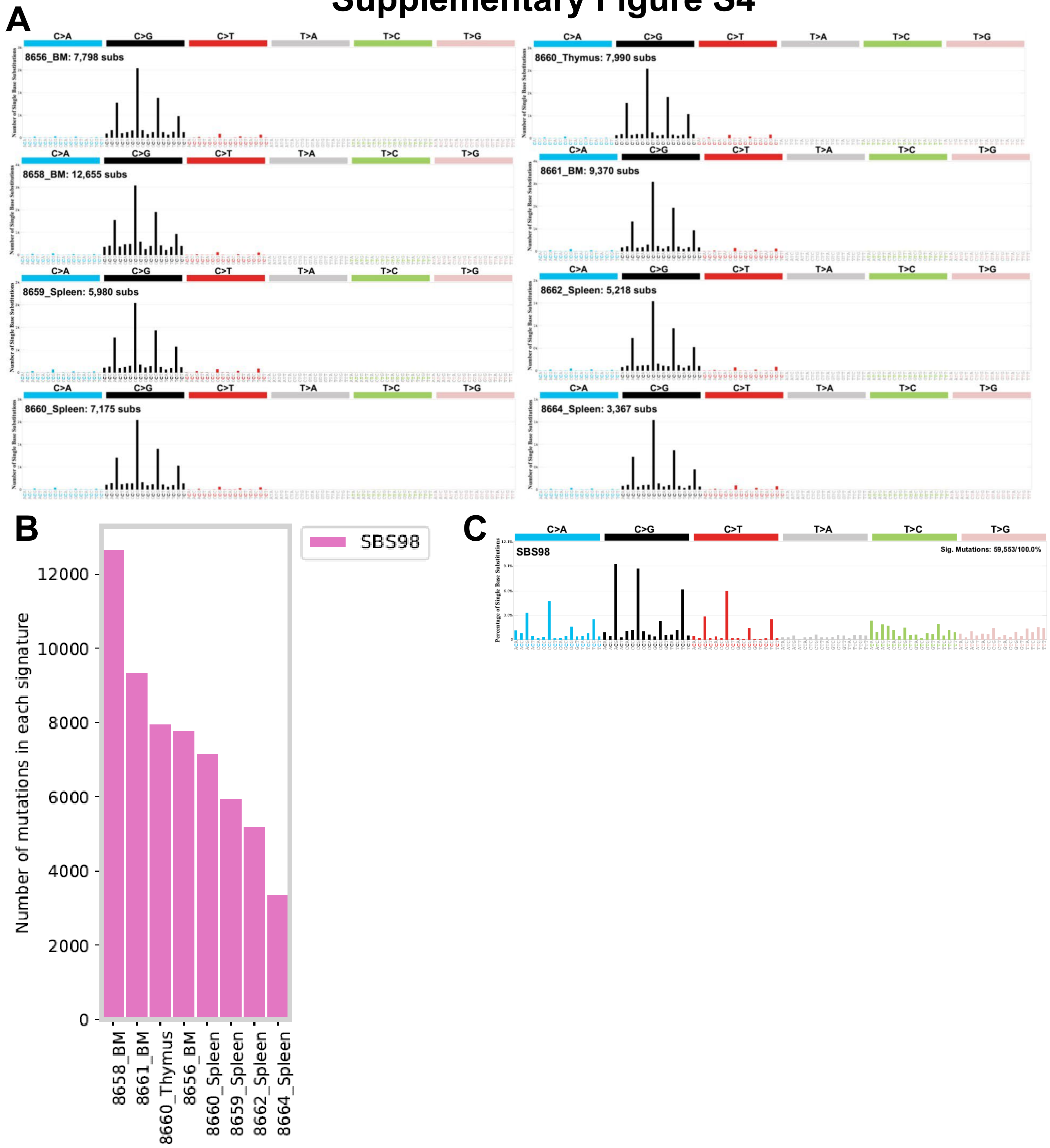

Supplementary Figure S4. Comparison of ATC induced mutational signature with known mutational signatures.

A. SBS plot for all RAG-1 KO BCP ALL (7) and RAG-1 KO T-ALL (1). **B.** An assignment solution activity plot showing the number of mutations in each signature on the y-axis and the sample name on the x-axis. Only SBS98 was identified. **C.** Assignment solution showing SBS 96 plot for the signature identified (SBS98; 100%).

Supplementary Figure S5

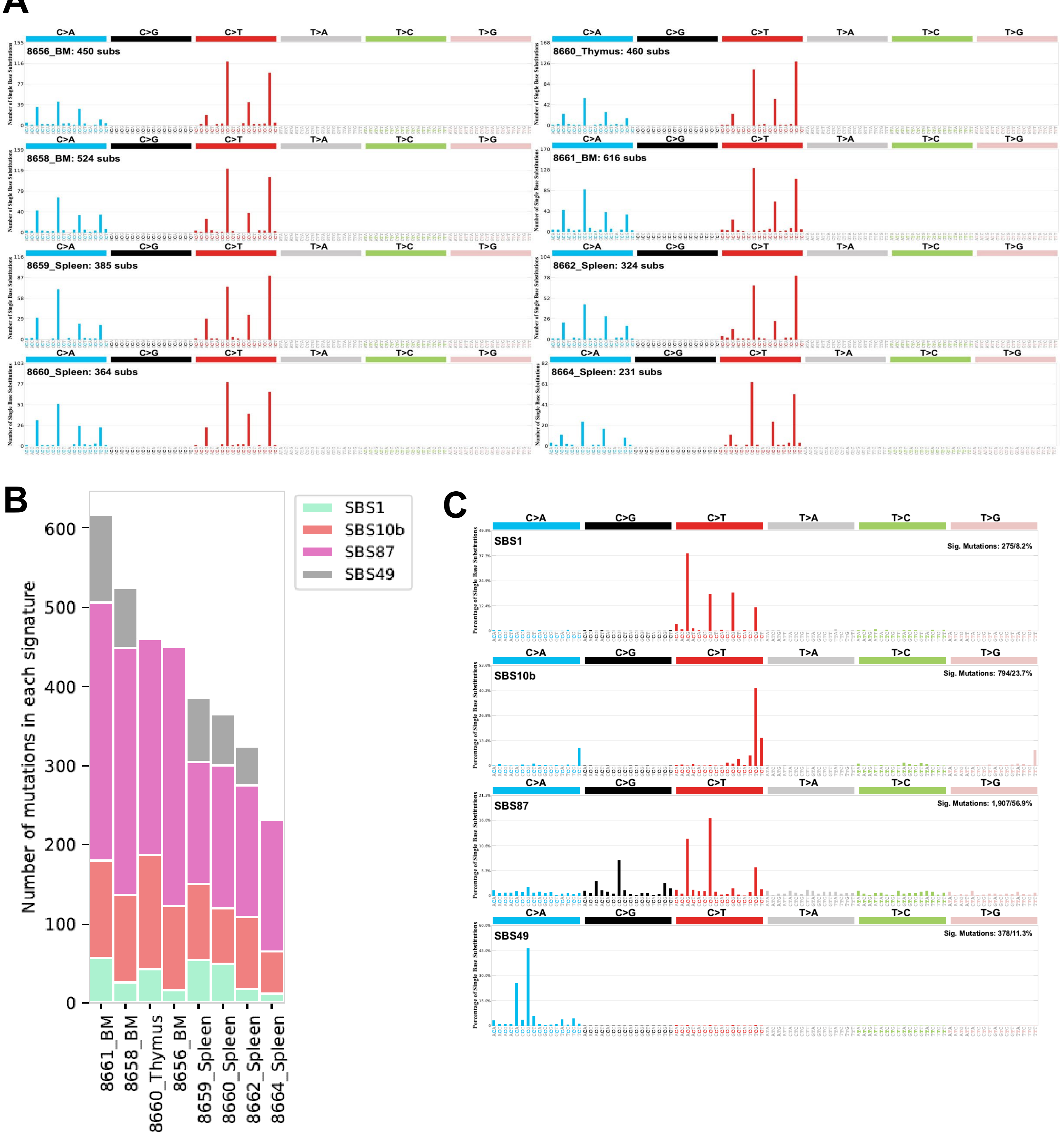

Supplementary Figure S5. Comparison of ATC induced C>T and C>A mutations mutational signature with known mutational signatures.

A. B. SBS plot of RAG-1 KO leukemia samples for C>T and C>A mutations. C>G mutations were removed from the data set to allow visualization of non-C>G mutations. **B.** Assignment solution activity plot showing the number of mutations in each signature on the y-axis and the sample name on the x-axis. When using only C>T and C>A mutations from RAG-1 KO BCP ALL (7) and RAG-1 KO T-ALL, SBS1, SBS10b, SBS87 and SBS49 mutational signatures were identified, with SBS87 being the most prominent. **C.** Assignment solution showing SBS 96 plot for signature identified with the proportion of mutation types for that signature.

Supplementary Figure S6

A

Recurrent C>G at CpG

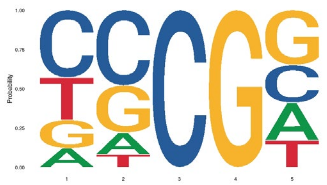

5 nucleotide consensus-sequence for recurrent C>G mutations at CpG sites

	position1	position2	position3	position4	position5
A	96(0.12)	115(0.14)	0	0	205(0.24)
C	357(0.43)	412(0.48)	844	0	193(0.24)
G	154(0.18)	250(0.3)	0	844	307(0.35)
T	237(0.27)	67(0.08)	0	0	139(0.17)

Non-recurrent C>G at CpG

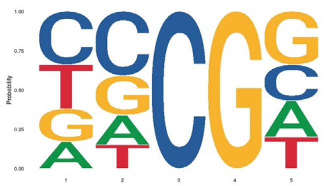

5 nucleotide consensus-sequence for non-recurrent C>G mutations at CpG sites

	position1	position2	position3	position4	position5
A	6677(0.17)	7878(0.19)	0	0	8995(0.23)
C	13255(0.34)	16216(0.41)	39170	0	9141(0.23)
G	8375(0.21)	9747(0.25)	0	39170	13115(0.34)
T	10863(0.28)	5329(0.15)	0	0	7919(0.2)

B

All C>G at non-CpG

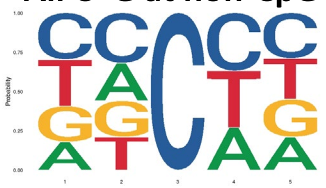

5 nucleotide consensus-sequence for C>G mutations at non-CpG sites

	position1	position2	position3	position4	position5
A	2752(0.176)	3742(0.239)	0	4246(0.271)	3346(0.214)
C	4660(0.298)	4998(0.319)	15661(1.0)	5713(0.365)	4536(0.29)
G	3578(0.228)	3634(0.232)	0	0	3773(0.241)
T	4671(0.298)	3287(0.21)	0	5702(0.364)	4006(0.256)

C

All samples	CpG site	non-CpG site	CpG/non-CpG
C>G Count	40014	15661	**2.6**
C>T Count	1983	132	**15.02**
C>A Count	1067	170	**6.3**

Supplementary Figure S6. Mutation signatures of individual mouse leukemia samples following exposure to ATC. **A.** Sequence logo and position frequency for 5 nucleotide context for recurrent and non recurrent C>G mutations at CpG dinucleotides based on all samples. Frequency of each base is shown in brackets next to absolute count. **B.** Sequence logo and position frequency for 5 nucleotide context for C>G mutations at non-CpG dinucleotides. **C.** Absolute count and ratio of all C>G , C>T and C>A mutations at CpG and non-CpG dinucleotides for all samples.

Supplementary Figure S7

A

C>T mutations		Diagnosis	BCP-ALL							T-ALL
		Immunophenotype	CD19+B220+	CD19+B220+	CD19+B220+	CD19+B220+	CD19+B220+	CD19+B220+	CD19+B220+	CD8+
		Genotype	Rag1KO							
	chr	WES sample no.	**8656_BM**	**8658_BM**	**8659_Spleen**	**8660_Spleen**	**8661_BM**	**8662_Spleen**	**8664_Spleen**	**8660_Thymus**
**B-cell differentiation genes**	X	**Bcor**								
	11	**Ikzf1**								
	11	**Ebf1**								
	15	**Ep300**								
	4	**Pax5**								
	16	**Runx1**								
	6	**ETV6**								
	10	**Tcf3**								
**Tyrosine Kinase signaling**	8	**Jak3**								
	4	**Jak1**								
	5	**Flt3**								
	2	**Abl1**								
	15	**Il7r**								
	9	**Cbl**								
**Ras/Rho signalling**	5	**Ptpn11**								
	6	**Kras**								
**Trp53 pathway**	11	**Trp53**								
**Chromatin modifiers**	9	**Kmt2a**								
	9	**Setd2**								
	16	**Crebbp**								
	X	**Atrx**								
	15	**Arid2**								
	2	**Asxl1**								
	X	**Kdm6a**								

	missense mutation
	stop gained
	splice acceptor

B

C>A mutations		Diagnosis	BCP-ALL							T-ALL
		Immunophenotype	CD19+B220+	CD19+B220+	CD19+B220+	CD19+B220+	CD19+B220+	CD19+B220+	CD19+B220+	CD8+
		Genotype	Rag1KO							
	chr	WES sample no.	**8656_BM**	**8658_BM**	**8659_Spleen**	**8660_Spleen**	**8661_BM**	**8662_Spleen**	**8664_Spleen**	**8660_Thymus**
**B-cell differentiation genes**	X	**Bcor**								
	11	**Ikzf1**								
	11	**Ebf1**								
	15	**Ep300**								
	4	**Pax5**								
	16	**Runx1**								
	6	**ETV6**								
	10	**Tcf3**								
**Tyrosine Kinase signaling**	8	**Jak3**								
	4	**Jak1**								
	5	**Flt3**								
	2	**Abl1**								
	15	**Il7r**								
	9	**Cbl**								
**Ras/Rho signalling**	5	**Ptpn11**								
	6	**Kras**								
**Trp53 pathway**	11	**Trp53**								
**Chromatin modifiers**	9	**Kmt2a**								
	9	**Setd2**								
	16	**Crebbp**								
	X	**Atrx**								
	15	**Arid2**								
	2	**Asxl1**								
	X	**Kdm6a**								

	missense mutation
	stop gained
	splice acceptor

Supplementary Figure S7. C>A and C>T mutations induced by ATC treatment of RAG-1 KO mouse.
A. Plot showing B-lineage ALL associated C>T acquired SBS mutations in ATC treated RAG-1 KO leukemia samples.
B. Plot showing B-lineage ALL associated C>A acquired SBS mutations in ATC treated RAG-1 KO leukemia samples.

Supplementary Figure S8

A

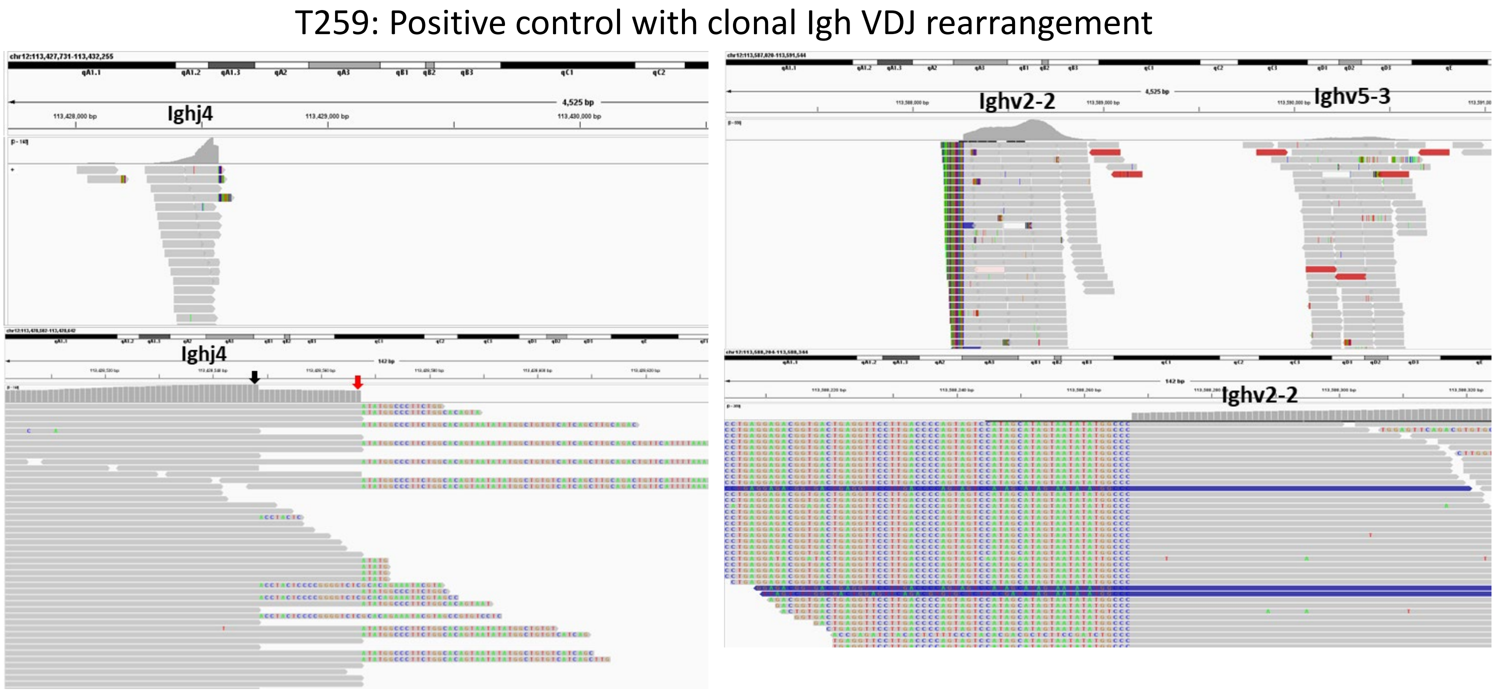

B

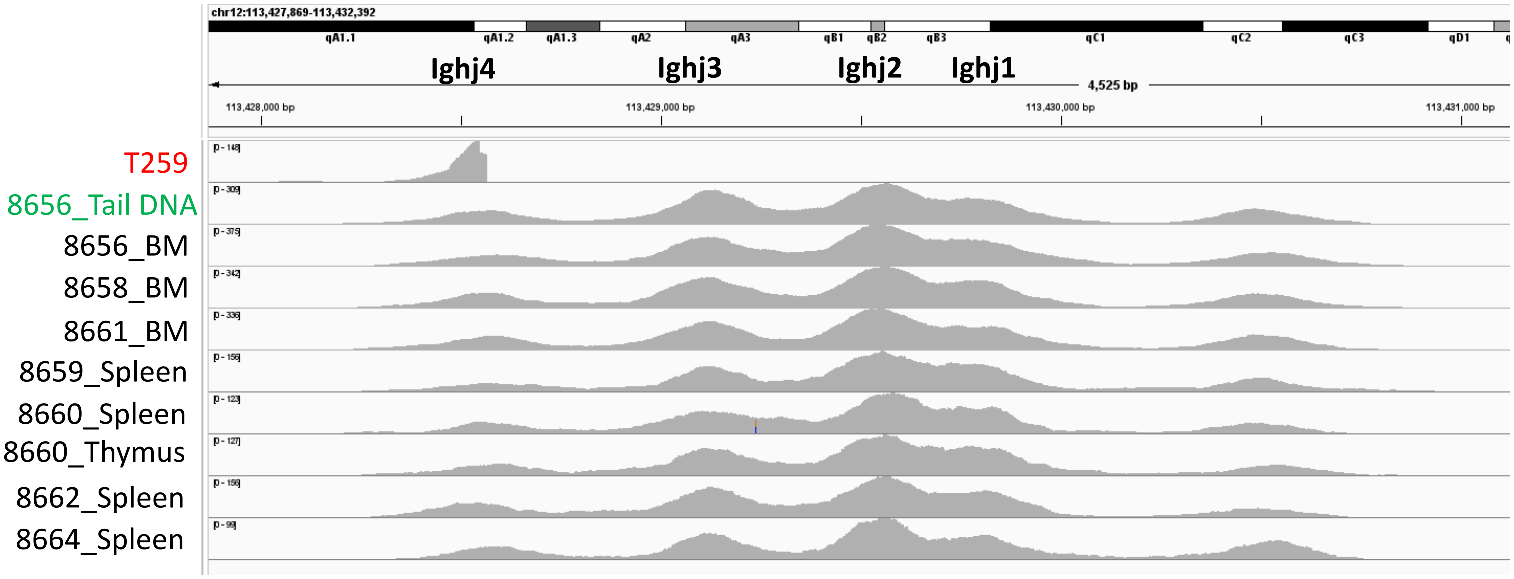

Supplementary Figure S8. RAG-1 KO BCP-ALL do not undergo clonal *Igh* VDJ rearrangement.
A. Whole exome sequencing reads for T259 cells (murine BCP-ALL cell line) shown as positive control for clonal VDJ recombination between *Ighj4* and *Ighv2-2*. Black and red arrow represent two distinct VDJ junctions involving the *Ighj4* segment, one for each allele. A single *Ighv2-2* junction is shown. **B.** Whole exome sequencing reads for RAG-1 KO BCP-ALL samples showing absence of *Igh* VDJ recombination for all four *Ighj* segments (*Ighj1-4*). T259 is used as positive control for clonal VDJ recombination and shows deletion of *Ighj1-3* due to recombination involving *Ighj4*; 8656 tail sample is used as negative control for *Igh* VDJ recombination.

Supplementary Figure S9

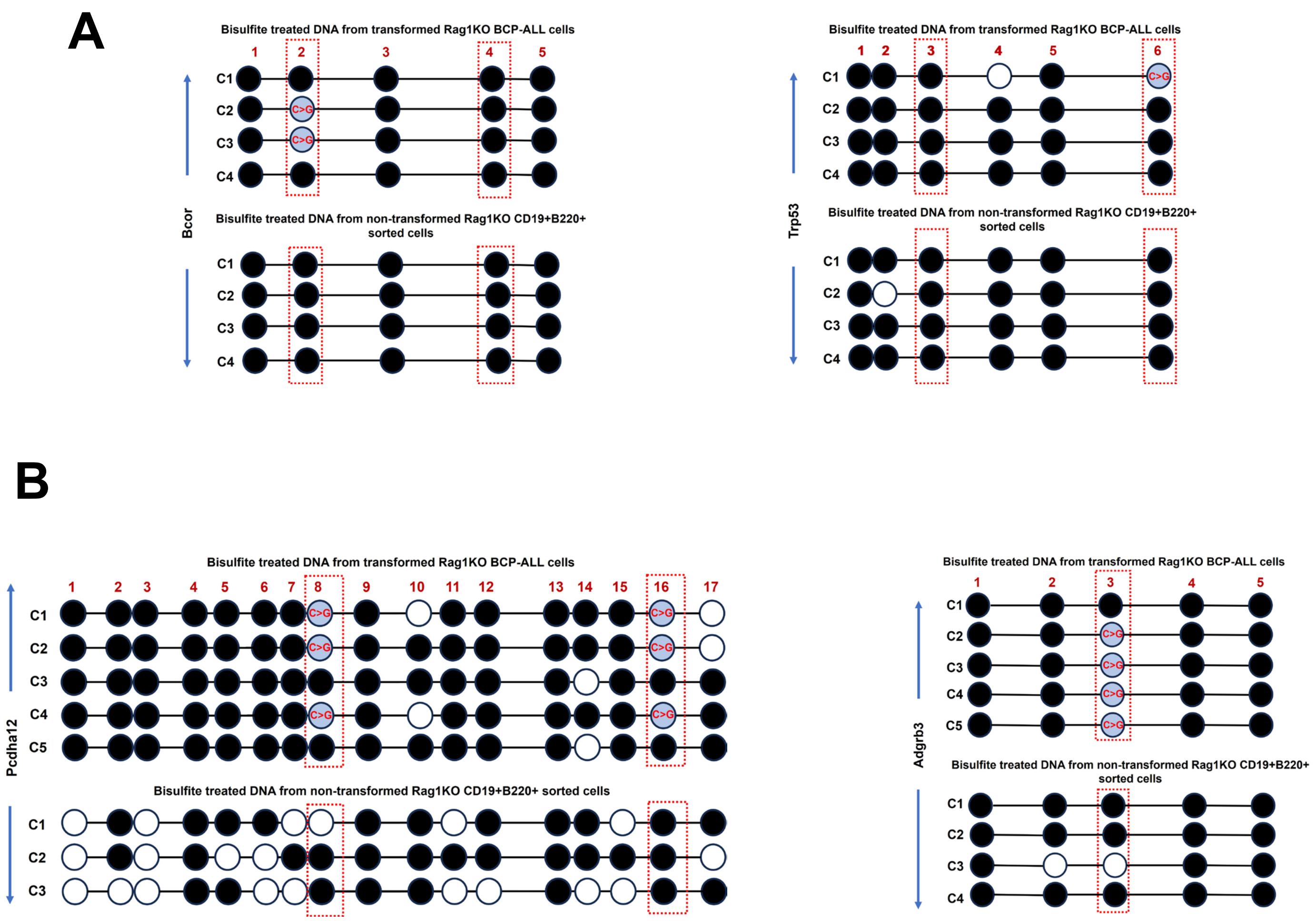

Supplementary Figure S9. CpGs with C>G transversion in RAG-1 KO ATC-BALL tumor samples are highly methylated.
A. Methylation status of C>G mutated CpG's at Bcor and Trp53 loci using RAG-1 KO ATC leukemia sample and untreated RAG-1 KO CD19+B220+ sorted cells. **B.** Methylation status of C>G mutated CpG's at *Pcdha12* and *Adgrb3* loci using RAG-1 KO ATC leukemia sample and untreated RAG-1 KO CD19+B220+ sorted cells

Supplementary Figure S10

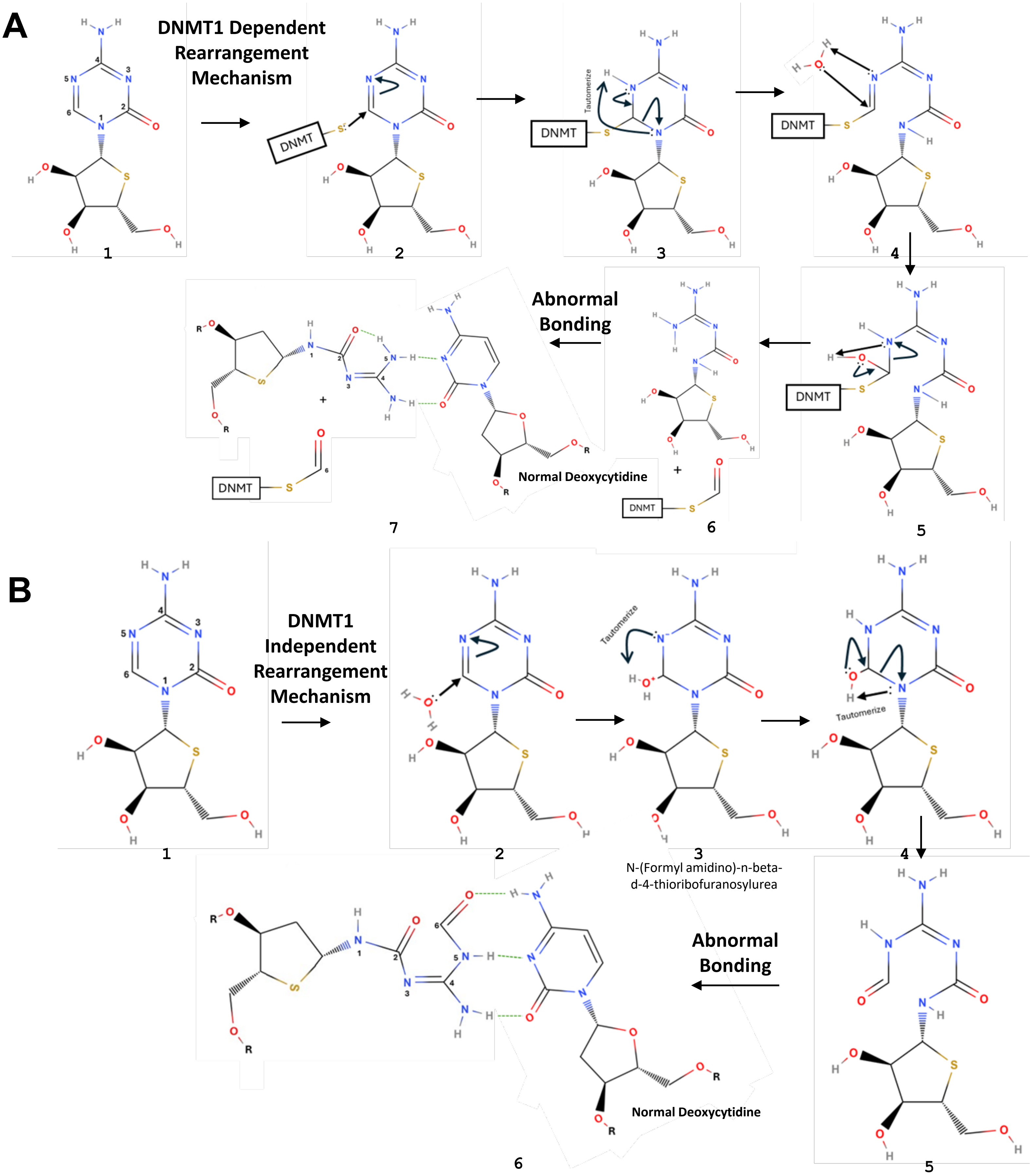

Supplementary Figure S9. Proposed model of ATC induced single base substitution mechanism.
A. Proposed model for DNMT1 dependent ATC induced single base substitution mechanism. **B.** Proposed model for DNMT1 independent ATC induced single base substitution mechanism.

Supplemental Methods

Mouse strain

RAG-1 KO mice (B6.129S7-RAG-1tm1Mom/J) were purchased from The Jackson Laboratory. All animal experiments were approved by the NCI Intramural Animal Care and Use Committee and maintained in an NIH intramural animal facility.

Drug treatment and schedule

5-Aza-4'-thio-2'-deoxycytidine (Aza-TdCyd or ATC, NSC777586—CAS 169514-76-5) was obtained from the Drug Synthesis and Chemistry Branch, Developmental Therapeutics Program (RRID:SCR_003057), Division of Cancer Treatment and Diagnosis, NCI. ATC stock solutions were prepared as described previously[1]. In brief, stock solutions of 1.169 umol/L were prepared and stored as aliquots at minus 20°C. Aliquots were thawed and used once and not refrozen. For in vivo treatment RAG-1 KO mice were treated with ATC (1mg/kg) or PBS (as control) once daily via intraperitoneal injection for 5 days per week for 2 weeks followed by 1-week rest and repeating this cycle again till the end point of treatment (euthanasia of moribund mouse or end of experiment).

Leukemia assessment

Mice were monitored clinically for signs of illness such as tachypnea, lethargy, kyphosis, and ruffled fur. Complete blood count (CBC) using peripheral blood from tail vein was obtained pre-treatment and on moribund mice when feasible. Moribund mice were euthanized and evaluated for leukemia using established guidelines[2]. Moribund mice were euthanized via CO_2 asphyxiation. Mice (including mice that were found dead if feasible) were examined for physical signs of leukemia development including splenomegaly, hepatomegaly, hind leg paralysis and compression of the pelvic region. Tissue samples were collected from BM, spleen and thymus for immunophenotype analysis and nucleic acid isolation as described previously[1].

Flow cytometry

Flow cytometry was performed as described previously[1]. Single-cell suspensions were prepared from bone marrow, spleen, and thymus tissue and filtered through a 40uM mesh filter into HF2 (HBSS + 2%FBS). Cells were counted and resuspended in an HF2 solution containing 5% rat serum solution to block antibody binding to Fc receptors. Cells were then incubated with a cocktail of antibodies for 30 min at 4°C. The antibody cocktail included: Mac1-PE; (clone-M1/70); BioLegend, Gr1-APC-eFluor 780; (clone-RB6-8C5) ;Invitogen, CD19-AlexaFluor700; (clone-6D5); BioLegend, B220-APC-eFluor 780; (clone-RA3-6B2); eBioscience, B220-PerCP-eFluor710; (clone-RA3-6B2); Invitrogen, CD71-BV421; (clone-R17217); BDBiosciences, Ter119-APC; (clone-TER119); BioLegend, CD43-FITC; (clone-eBioR2/60); eBioscience, CD249(BP1)-APC; (clone-6C3); Invitrogen, CD24(HSA)-PE; (clone-30-F1); eBioscience, CD4-PerCP-Cy5.5; (clone-GK1.5); BioLegend, CD8-APC-Cy5.5; (clone-53-6.7); Abcam, 7AAD(viability); Biolegend. After incubation, cells were washed in PBS and resuspended in HF2. Cells were then analyzed using a three laser (Violet: 405nm, Blue: 488nm, and Red: 640nm) Cytek Northern Lights cytometer.

Flow cytometry sorting

Untreated RAG-1 KO or WT BM cells were harvested as described earlier. For sorting experiments RAG-1 KO or WT BM from three mice were pooled prior to sorting. Cells were stained as described above with an antibody cocktail that included: B220-FITC; (clone-RA3-6B2); BD Pharmingen, CD19-Alexa Fluor 700; (clone-6D5); BioLegend. After incubation cells were washed in PBS and resuspended in HF2 plus DAPI (viability); BD Pharmingen. Cells were then analyzed and sorted using a BD FACSAria IIu sorting instrument.

Bulk RNA sequencing

BM and spleen samples from leukemic RAG-1 KO mice samples and sorted RAG-1 KO BM samples were used for total RNA isolation as described previously[3]. Total RNA-Seq samples were pooled and sequenced on NovaSeq 6000 SP using Illumina® Stranded Total RNA Prep, Ligation with Ribo-Zero Plus and paired-end sequencing. Raw fastq reads were analyzed using Partek™ Flow™ software, v12.4.2. Analysis steps included base and adaptor trimming, STAR Alignment (using reference genome=mm10), quantification of aligned reads (using Partek E/M), and normalization of gene counts (using DESeq2(poscounts) + add0.01). Differential gene expression analysis was performed using DESeq2 method.

Hierarchical clustering analysis was done using R statistical software (v 4.4.1) and RStudio (v 2023.12.0.369) with base R function.

Gene set enrichment analysis

GSEA analysis was performed using GSEA software (Broad Institute v 4.3.2)[4,5]. Differential gene expression analysis between RAG-1 KO ALL samples and RAG-1 KO CD19+B220+ samples was performed using DESeq2 and genes with p<=0.05 and fold change <= -1.5 or >= 1.5 were used for enrichment analysis with gene sets curated from publicly available murine B lineage ALL data (GSE221597) and human B lineage ALL data (GSE79533). To curate mouse and human gene sets, GEO2R tool provided on Gene Expression Omnibus was used for performing comparisons between selected groups (leukemia and control) from GSE221597 or GSE79533.

Whole-exome sequencing

WES was performed on RAG-1 KO leukemia samples or B cell precursor (BCP) ALL cell line (T259) samples as described previously[1]. Briefly, genomic DNA (500 ng) was sheared to a mean size of 300 bp on a S2 Covaris Ultrasonicator. Illumina libraries were prepared using a KAPA HyperPrep Kit, according to the manufacturer's instructions, incorporating unique dual indexes (IDT). Mouse Illumina libraries were pooled and enriched using Agilent SureSelect XT System with Mouse All Exon baits according to the manufacturer's instructions. Enriched libraries were sequenced on an Illumina NextSeq 2000 Sequencing System using P3 (300 cycle) reagents. Data processing and variant calling procedure followed the Best Practices workflow recommended by the Broad Institute. Briefly, the raw sequencing reads were mapped to mouse genome build 10 (mm10) by the Burrows–Wheeler Aligner followed by local realignment using the GATK suite v4.2.5.0 9 from the Broad Institute, and the Picard tools marked duplicated reads. The somatic variants were called by MuTect2 within the GATK suite. The somatic variants were first filtered with the GATK recommended filtering criteria.

For SBS mutational signature analysis, somatic variants were further filtered by the following criteria: baitRegion = "TRUE," FILTER = "PASS," and AD_TUMOR >= 5. The filtered variants were used for the

Mutational Signature analysis, for which the software SigProfilerMatrixGenerator v1.2 and SigProfilerExtractor v1.1.4 from AlexandrovLab at UCSD were used.

For FoundationOne_Heme panel gene study, somatic variants were further filtered by the following criteria: baitRegion = "TRUE", FILTER = "PASS", AD_TUMOR >= 5, Annotation_Impact = "MODERATE or HIGH", AF_TUMOR>=0.2.

For in vitro T259 cells DNMT1 inhibitor assay (GEMINI assay clones), exome samples were pooled and sequenced on NovaSeq Xplus 1.5B run using Agilent SureSelect XT Mouse All Exon and paired-end sequencing mode. The samples were mapped, and variants were called using Dragen. Mapping was performed against reference genome mm10. Coverage statistics were also measured using Dragen. The mapped sequencing depth coverage over target (after alignment and marking duplicates) was between 153x to 200x. The mean insert size for these samples was between 184 and 210 bases. More than 98% of the target region have the coverage above 20x.

For SBS mutational signature analysis, somatic variants were further filtered by the following criteria: FILTER = "PASS", "PANEL OF NORMALS", and AD_TUMOR >= 5. The filtered variants were used for the Mutational Signature analysis, for which the software SigProfilerMatrixGenerator v1.2 and SigProfilerExtractor v1.1.4 from AlexandrovLab at UCSD were used.

Bisulfite treatment, bisulfite specific PCR and sequencing

Genomic DNA isolated from RAG-1 KO leukemia samples and sorted RAG-1 KO BM CD19+B220+ cells was bisulfite treated using an Qiagen EpiTect Bisulfite Kit (cat. #59104) according to manufacturer's protocol. Bisulfite PCR primers for amplification of bisulfite converted DNA amplicons were designed using MethPrimer tool[6]. PCR products were gel purified using Qiagen QIAquick Gel Extraction Kit (cat. #28706) and subcloned using TA cloning (Promega, cat. #A1360). Plasmid DNA isolated from positive subclones were sequenced at the NCI DNA Minicore to determine methylation status.

In vitro drug treatment (GEMINI assay)

In vitro DNMT1 inhibitor treatment of a murine BCP ALL cell line (T259) was performed using a Genotoxic Mutational Signature Identified After Clonal Expansion (GEMINI) assay, as described previously[1]. Briefly, an exponential growth culture of T259 cells was single cell sorted into 96 well plates. A single well was then expanded and treated in 25-ml flask with non-azanucleoside DNMT1 inhibitor, GSK-3685032 (GSK), ATC, or both GSK and ATC for 12 days, refreshing the media (IMDM complete media, 10ng/ml mIL-7, 10ng/mL β-mercaptoethanol) and DNMT1 inhibitors every 3 days. Following treatment, treated cells were single-cell sorted and expanded in 96-well plates. Three clones were then picked at random to expand for whole-exome sequencing (WES). Cell counts and viability were conducted using an automated cell counter (TC20 Automated Cell Counter, Bio-Rad) and Trypan Blue Viability assay (Lonza Bio- Whittaker Trypan Blue 0.4%, Thermo Fisher Scientific, cat. #BW17- 942E). In one experiment, two independent clones were present in one sample (50ATC_0GSK_C.5), as evidenced by a mean VAF of 0.2-0.3 instead of 0.4-0.6; this clone was treated as two individual clones.

Statistics and hypothesis testing

Survival curve data was generated using GraphPad Prism v8.4.3 (RRID: SCR_002798; GraphPad Software, LLC) and survival analysis significance was tested using Mantel-Cox log-rank test. Mann-Whitney U test

was used for testing significance when comparing mutation counts of different single base substitution mutations in RAG-1 KO mouse samples. Unequal variance t-test (Welch's test) was used for testing significance when comparing mutation counts of different single base substitutions from different clones from invitro GEMINI assay. Unpaired t test was used for testing significance when comparing normalized read counts of *Dck* mRNA from RNASeq samples. P values of <0.05 were considered statistically significant in the context of this study.

References

1. Bertoli RM, Chung YJ, Difilippantonio MJ, et al. The DNA Methyltransferase Inhibitor 5-Aza-4'-thio-2'-Deoxycytidine Induces C>G Transversions and Acute Lymphoid Leukemia Development. *Cancer Res*. 2024;84(15):2518-2532.
2. Morse HC, 3rd, Anver MR, Fredrickson TN, et al. Bethesda proposals for classification of lymphoid neoplasms in mice. *Blood*. 2002;100(1):246-258.
3. Goldberg L, Negi V, Chung YJ, et al. Mutant Idh2 Cooperates with a NUP98-HOXD13 Fusion to Induce Early Immature Thymocyte Precursor ALL. *Cancer Res*. 2021;81(19):5033-5046.
4. Subramanian A, Tamayo P, Mootha VK, et al. Gene set enrichment analysis: a knowledge-based approach for interpreting genome-wide expression profiles. *Proc Natl Acad Sci U S A*. 2005;102(43):15545-15550.
5. Mootha VK, Lindgren CM, Eriksson KF, et al. PGC-1alpha-responsive genes involved in oxidative phosphorylation are coordinately downregulated in human diabetes. *Nat Genet*. 2003;34(3):267-273.
6. Li LC, Dahiya R. MethPrimer: designing primers for methylation PCRs. *Bioinformatics*. 2002;18(11):1427-1431.
